# The effects of the Covid-19 pandemic on ecotourism, a study from West of Iran

**DOI:** 10.3389/fpubh.2022.983025

**Published:** 2022-09-06

**Authors:** Mehdi Rahimian, Mandana Masoudi Rad, Hossein Zareei

**Affiliations:** ^1^Department of Agricultural Economics and Rural Development, Faculty of Agriculture, Lorestan University, Khoram Abad, Iran; ^2^Department of Geography and Urban Planning, University of Sistan and Baluchestan, Zahedan, Iran; ^3^Department of GIS, Islamic Azad University North Tehran Branch, Tehran, Iran

**Keywords:** ecotourism, sustainable tourism, COVID-19, fuzzy Delphi, Iran

## Abstract

Most tourism researchers agree on the effects of the COVID-19 on ecotourism. The present study aims to assess the effects of the COVID-19 pandemic on the ecotourism status in Lorestan province in Iran. To this aim, 29 effects were identified using Delphi technique. According to results, the effects were divided into six categories including the decreased number of incoming tourists, the reduced activity of hotels and resorts, the declined income of goods and service suppliers for tourists, the decreased activity of travel agencies and tourist tours, as well as positive and negative environmental effects. Generally, the results provide new knowledge in the field of ecotourism crisis management. In addition, the identified effects provide the basis for further research on the method of reducing the negative effects.

## Introduction

The tourism industry has a special place in the economies and plays a significant role as a stimulus in the economic (1, 2), social, and cultural structure, especially in developing countries (3). According to the experts, the world is experiencing the fourth industrial revolution, despite tourism, in which the physical, digital, and biological domains are integrated (4).

However, tourism destinations have always been affected by numerous crises such as climate change, natural disasters, recession, political instability, internal turmoil, and terrorism (5, 6), which have had adverse effects on tourism and its industry. The pandemic of the COVID-19 is considered as a new crisis with great effect on a large number of sectors, especially tourism (7), which has been regarded as the biggest victim of the pandemic (8). This pandemic is the worst global epidemic with the greatest effect on the world after World War II (9). Some believe that the COVID-19 has affected the tourism sector more than any other previous diseases (10).

Ecotourism is a type of nature-based tourism that does not have the negative environmental, economic and social effects associated with mass tourism (11). This type of tourism includes cultural and environmental awareness, environmental protection, and empowerment of local and host communities (12). Ecotourism has been hailed as a new opportunity for developing countries because it includes responsible trips to nature which preserve the environment and improve the lives of local people (13, 14). Ecotourism destinations are one of the profitable and active subcategories in the tourism industry (15). These destinations offer services to tourists interested in nature-based tourism due to their natural potential (16).

The unpleasant consequences of the Covid-19 pandemic on most businesses and the various problems it has created for them are undeniable. Among these businesses are the activities of ecotourism destinations, which have faced many destination challenges (17–20). This epidemic has caused the reduction of activity or the complete closure of many ecotourism destinations (16). The results of an online study examining the effects of the COVID-19 pandemic on ecotourism-protected areas show that in 38 African countries, the pandemic has caused a significant reduction in destination visits, local livelihoods, and logistics, conservation, and environmental services (21). This pandemic has caused more restrictions on travel to ecotourism destinations and as a result, reduced income from this sector. The decrease in income has also led to a reduction in conservation budgets and, as a result, an increase in poaching (22). The COVID-19 continues to have positive and negative environmental consequences in the ecotourism sector, despite its negative economic effects. Positive consequences have emerged from the restrictions on human contact and industrial activity, while negative ones increased poaching, wildlife trafficking, and deforestation following declining tourism revenues in indigenous communities (12).

The points mentioned above, as well as the literature review in the next section, show that most of the research related to the effects of Covid-19 on ecotourism is focused on the environmental aspect and the protection of ecotourism areas. This focus is due to the principles of sustainability in ecotourism goals. Few studies have simultaneously examined the economic, social, and environmental effects of Covid-19 on ecotourism. Therefore, the necessity of conducting a study that comprehensively examines all the effects was felt. In this regard, there are some basic question. In addition to biological effects, what other effects has covid-19 had on the ecotourism industry? What is the state of residences, restaurants, tourist tours, and travel agencies during the Corona virus? To what extent has the volume of tourists entering tourist areas decreased? And to what extent has the employment of ecotourism sector activists been damaged? The present research is an attempt to fill the indicated research gap and answer the proposed questions.

Despite having diverse and valuable attractions, ecotourism in Iran did not have favorable conditions before the Covid-19 pandemic. After the epidemic, its situation has become much more unfavorable (19). One of the ecotourism areas of Iran is Lorestan province. Lorestan province, which is located in the west of Iran has a variety of climatic characteristics due to its position in the middle part of Zagros and having extensive mountainous areas, high peaks, valleys, and scattered plains with a special geographical location influenced by two aerial fronts of the Mediterranean and Indian Ocean. The special geographical location of the province has led to the formation and attraction of huge tourists, especially in the field of ecotourism in the province. So that before the outbreak of covid-19, about 1450,000 people visited the ecotourism areas of Lorestan province (23). The ecotourism industry, which is among the most significant sources of income for the province (non-governmental economic source) and a source of direct and indirect income for a large number of residents in the province, is now completely affected by the COVID-19, resulting in losing its sources of income. Many ecotourism destinations in Lorestan province and the effects of Covid-19 on the activities of these destinations are the reasons for choosing this province and this topic for research in this province. The present study aims to evaluate the effect of the COVID-19 on the ecotourism industry in Lorestan province.

Some of the main characteristics of this study are:

- Understanding the effects of Covid-19 on the ecotourism industry from various economic, social, and environmental aspects.
- Obtaining experts' opinions according to the fuzzy Delphi technique used in the research.
- Providing solutions for managing ecotourism destinations in crisis conditions.

### Literature review

Many studies have been conducted on the effects of the COVID-19 on tourism because of the importance of the issue.

The presence of mass tourists in natural destinations is not always in favor of ecotourism. In confirmation of this claim Lecchini et al. (24) studied the impact of human activity at France reef eco-tourism sites. They found that fish density considerably increased in the absence of tourists. So, their research focuses on the influence of tourists on fish communities.

Rosselló et al. (25) examined the risk of infectious diseases and international tourism demand and indicated that the eradication of diseases such as malaria, yellow fever, and Ebola in affected countries has increased about 10 million tourists in all over the world. Thus, the quantitative aspect of health policy outcomes should be considered given the strategic significance of the tourism sector in a large number of countries. In other words, the eradication of diseases affects the development and sustainability of tourism both directly and indirectly. In addition, Cherkaoui et al. (26) argued that Moroccan ecotourism has been severely damaged following the COVID-19 pandemic. Illegal hunting, wildlife smuggling, and deforestation have been resumed due to the loss of income from ecotourism in rural communities. Further, Amador-Jiménez et al. (27) claimed that the presence and mobility of armed groups and the number of fires in the forests of Colombia has increased as the result of the quarantine and restrictions imposed by the COVID-19 pandemic. Researchers have attributed the situation to declining oversight by government and forest protection agencies during the pandemic. Furthermore, Lendelvo et al. (28) investigated the effect of the COVID-19 on community-based environmental protection and reported that it has affected the environment by disrupting the management and regular operational process of conservation, patrolling and wildlife monitoring, income and cash flow in conservation business operations, equity participation, job opportunities and local livelihood, community development projects and social benefits, budgeted projects and programs, and technical capacity resulting from communication technologies and equipment. According to Stone et al. (29), the tourism sector has stopped altogether with the spread of Covid-19 in Botswana. This trend has promoted ecotourism for domestic tourists and thus has a negative impact on the protection of ecotourism destinations. COVID-19 has also affected community development through sudden loss of jobs and income. Although several positive environmental effects have also been experienced. In another study, Buckley (30) asserted that the ecological effects of reduced tourism vary depending on the level of development in countries since the number of visitors and related environmental impact has greatly fallen in the developed countries, particularly in the wildlife, despite the continued public budgets and park conservation, resulting in providing appropriate opportunities for the successful reproduction of endangered species. However, the COVID-19 has led to adverse environmental effects such as poaching of endangered species, reduced environmental costs, deforestation, and the like in developing countries, where the cost of environmental protection is provided by tourism revenues, NGOs, and the like. According to Foo et al. (31), the COVID-19 has canceled a vast number of tours in Malaysia and reduced the number of tourists to this country significantly. In addition, current travel bans and sharp declines in demand around the world have put some airlines at risk of bankruptcy. A total of 170,084 hotel room reservations were canceled during 11 January-16 March 2020, leading to a loss of RM 68,190,364, which was directly attributed to the COVID-19 pandemic. Jovanović et al. (12) declared the positive consequences of the COVID-19 pandemic on ecotourism have outweighed the negative ones since restrictions on human contact and industrial activities have had positive environmental consequences. Nonetheless, other researchers believe that COVID-19 has affected ecotourism negatively, especially the wildlife conservation due to declining incomes and unemployment. In addition, Goretti et al. (32) provided some solutions to improve the tourism status in Asia and the Pacific during the COVID-19 pandemic, the most significant of which include strengthening health systems, changing the direction of sustainable tourism models, investing in new technologies, diversifying economic investments to avoid dependence on a single sector (tourism), investing in the manufacture or preparation of vaccines, considering long-term solutions for the restoration of tourism in accordance with the economy and environment, paying attention to travel health information such as compliance with health protocols by travelers, health insurance coverage, need for pre-travel viral tests, and the like. The results of an interesting research in Taiwan show that the arrival of foreign tourists and the departure of Taiwanese tourists are limited. Therefore, people have turned to outdoor leisure activities, especially lagoon tourism. In this research, Wu et al. (33) showed that despite the reluctance of Taiwanese people, the outbreak of the coronavirus has increased their interest in ecotourism. Mudzengi et al. (34) reported negative overall impacts of the COVID-19 pandemic on ecotourism. These effects include a decline in ecotourism visits, an increase in poaching, a global economic slowdown, and increased lobbying for more international hunting and wildlife trade.

Some studies have also provided solutions to reduce the effects of Covid-19 on ecotourism. Hosseini et al. (14) believe that it is very important to find solutions for affected businesses during the coronavirus pandemic. Based on this research the standardization of the centers and estimating demand numbers, have the main role in recovering ecotourism businesses in pandemic conditions. Gabriel-Campos et al. (35) emphasize enhancing the Local community's resiliency against the pandemic and accelerating impacts of climate change risks. They believe that the eco-tourism system and the relationship that the community has built with other local organizations have created sufficient tools to adapt to the effects of environmental crises. Komasi et al. (19), believe that five items are necessary to the development of nature-based tourism in Iran: national, regional, and global safety; economic stability; private sector participation; human crises such as diseases, wars, etc. national and international advertising, and travel costs. According to Mudzengi et al. (36), livelihood diversification, extension of domestic visits, aggressive marketing, capacity building, lobbying for government support, extension of effective interactions with stakeholders and developing an international hunting code of ethics are some of the strategies to adoption with pandemic shocks.

As shown, the COVID-19 has affected tourism and ecotourism in the economy negatively, resulting in reducing the tourists, travels, ticket sales for travel agencies, residence in hotels and resorts, food for restaurants, and direct income from tourism-related jobs. In addition, the COVID-19 has affected the environment in ecotourism areas, resulting in decreasing the supervision of environmental organizations during the pandemic and creating more opportunities for poachers and illegal forest exploiters to inflict more damage to the environment of plant and animal species in the absence of guards. Nevertheless, all of the COVID-19 effects have not been negative and a series of positive events have occurred in the environment sector due to the lower presence of tourists in ecotourism destinations during the pandemic, resulting in giving the opportunity to revive nature. In addition, fewer trees and shrubs have been cut down for tourism activities and cooking for tourists.

## Methodology

### Study area

**Figure 1:**
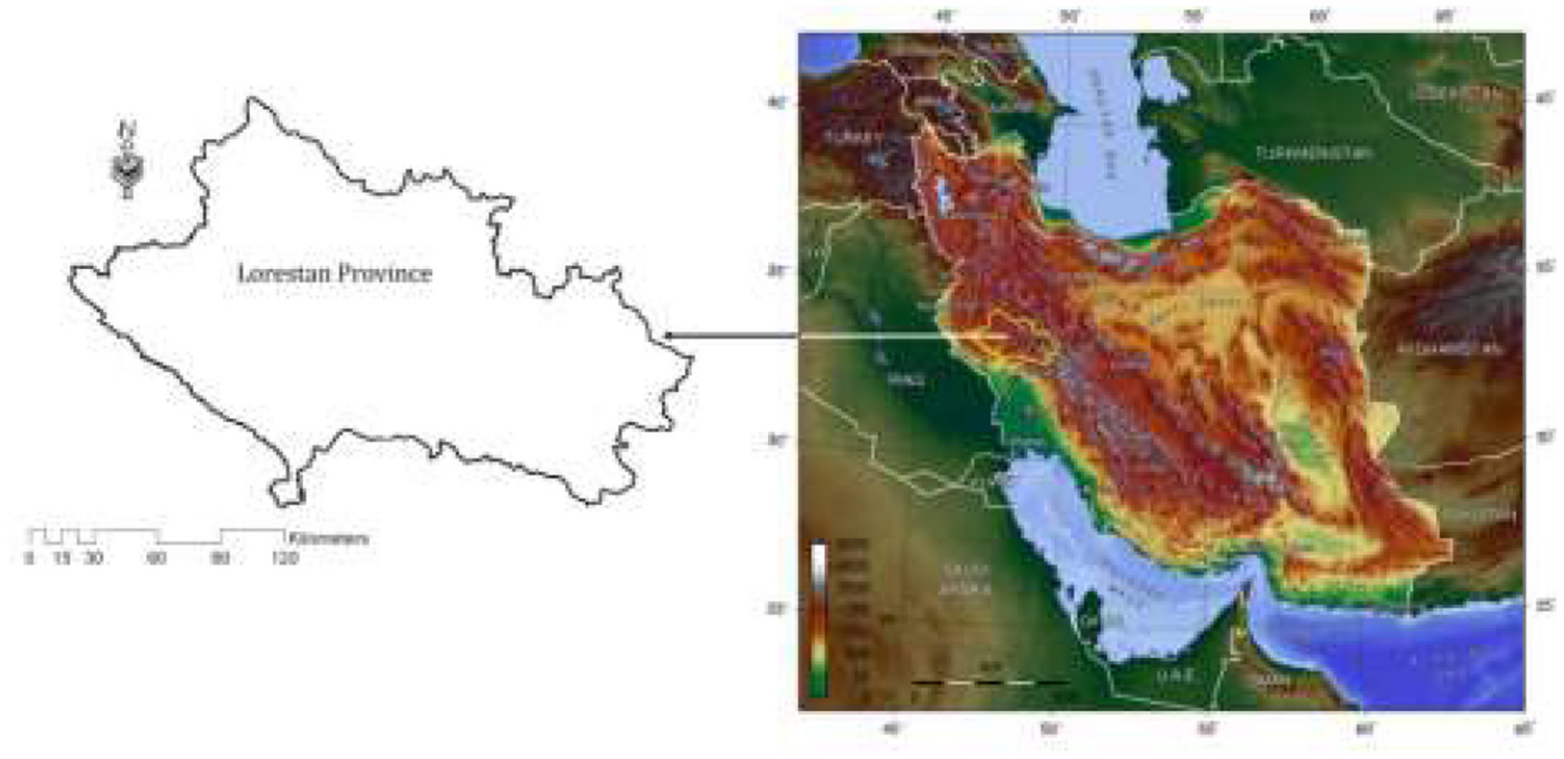
Survey locations for the study (38).

This study conducted in 2020 in the west of Iran. This study carried out in Khorramabad County, Lorestan Province, West of Iran, located between latitudes 32° 30' and 48°1' N and longitudes 55°17' and 61° 15' E. The total area of the province is 28064 km^2^ (37). The Figure 1 displays the location of Lorestan province in Iran. Lorestan province is known as the land of waterfalls, with Iran's most famous waterfalls being located in this province. Some waterfalls of Lorestan province, such as Bishe, Nojyan, Gerit, Vark, and Absefi host many tourists throughout the year. Bisheh waterfall is more popular with tourists than other waterfalls due to its beauty. So that it is known as the “bride of Iranian waterfall” (38). It is the reason for choosing this area as the study area in this research.

### Research design

The present study is regarded as applied and qualitative-quantitative with an exploratory approach in terms of objective and paradigm, respectively. This study was performed utilizing the Delphi method in two consecutive stages including during and before the Delphi stage. The data were analyzed by SPSS software. The Delphi method includes the following stages:

### The stage before the Delphi stage

In order to identify the markers, the effects of the COVID-19 pandemic on the ecotourism status in different parts of the world were identified and extracted through a library review and literature review. Then, keywords such as tourism, ecotourism, COVID-19, and the like were applied during searching for resources online, resulting in identifying a collection of 37 effects.

### The stage of Delphi stage

The Delphi method was used to identify other effects of the COVID-19 pandemic on ecotourism status in the study area and determine their significance. The Delphi method was developed as one of the constructed methods for reaching consensus in the RAND Company during the 1950s (39). The Delphi technique can be utilized to “identify” and “screen” the most significant decision-making indices. Thus, such technique is often applied to screen indices or reach a consensus on the significance of decision-making indices before using multi-criteria decision-making techniques although it is not considered as a multi-criteria decision-making method (40). Delphi studies usually end in two or three stages (41). In the present study, three consecutive rounds were followed to complete the consensus process. Selecting the experts in the Delphi process affects the quality of study and members in the panel of experts should have sufficient knowledge and awareness about the subject (40). The stage of Delphi (stage b) includes the following steps:

In the present study, academic and executive experts in the field of ecotourism, natural resources, environment, tourism, and NGOs active in ecotourism and tour leaders were utilized according to the subject. To this aim, 22 participants were selected based on the purposive non-probability sampling considering that the Delphi method can be applied with at least three people (42), which are regarded as sufficient. Table 1 shows the characteristics of the experts and their participation in the Delphi rounds.

**Table 1:** Characteristics of experts and their participation in rounds of the Delphi method.

**No**	**Gender**	**Age**	**Job**	**Organization**	**Participation in Delphi rounds**		
					**1**	**2**	**3**
1	Male	33	Private sector employee	Tourism NGO	**√**	**√**	**√**
2	Female	45	Government employee	Department of Tourism and Cultural Heritage	**√**	**√**	**√**
3	Male	46	Government employee	Department of Natural Resources	**√**	**×**	**√**
4	Male	38	Faculty member	University	**√**	**√**	**×**
5	Male	55	Government employee	Department of Tourism and Cultural Heritage	**√**	**√**	**√**
6	Male	39	Private sector employee	Tourism NGO	**√**	**√**	**√**
7	Male	44	Government employee	Department of Natural Resources	**√**	**×**	**√**
8	Female	42	Private sector employee	Tourism NGO	**√**	**×**	**√**
9	Female	48	Private sector employee	Tourism NGO	**√**	**√**	**×**
10	Male	53	Government employee	Environment Department	**√**	**√**	**√**
11	Male	40	Tour Leader	Travel agency	**√**	**√**	**√**
12	Male	33	Government employee	Department of Natural Resources	**√**	**√**	**×**
13	Male	35	Faculty member	University	**√**	**√**	**√**
14	Female	28	Tour Leader	Travel agency	**√**	**√**	**×**
15	Male	50	Government employee	Environment Department	**√**	**√**	**√**
16	Male	49	Government employee	Department of Tourism and Cultural Heritage	**√**	**√**	**√**
17	Female	39	Tour Leader	Travel agency	**√**	**√**	**√**
18	Male	53	Faculty member	University	**√**	**√**	**×**
19	Male	58	Government employee	Department of Tourism and Cultural Heritage	**√**	**√**	**√**
20	Female	43	Tour Leader	Travel agency	**√**	**×**	**√**
21	Female	51	Private sector employee	Tourism NGO	**√**	**√**	**√**
22	Female	29	Tour Leader	Travel agency	**√**	**√**	**√**

√ Participation in each round and × Non-Participation in each round.

In the next step, a questionnaire was designed with the feedback of the previous stage, which included the effects of the COVID-19 pandemic on the ecotourism status. The questionnaire was applied based on the Likert scale with five-point including extremely low, low, medium, high, and extremely high to assess the agreement of the panel members.

Finally, Kendall rank correlation coefficient was used to evaluate the consensus on the components and measure their significance. Kendall rank correlation coefficient is regarded as a scale to determine the degree of coordination and success between several ranks categories related to the number of individuals. Kendall rank correlation coefficient is regarded as a scale to determine the degree of coordination and success between several ranks categories related to the number of individuals (Table 2). The Kendall rank correlation coefficient shows the consensus between the individuals who categorize several categories according to their significance (43). Kendall correlation is obtained by Equation (1).


(1) $$W=\frac{s}{\frac{1}{12}k^{2}(N^{3}-N)}$$

where, $s=\sum {\left(R_{j}-\frac{\sum R_{j}}{N}\right)}^{2}$ = The sum of the squares (SOS) of *R*_*j*_ deviations from the average of *R*_*j*_s

*R*_*j*_ = Total rankings related to a factor

K = Number of ranking collections (number of experts)

N = Number of factors ranked

$\frac{1}{12}k^{2}\left(N^{3}-N\right)$ = Maximum SOS of *R*_*j*_ derivations from the average of *R*_*j*_s

**Table 2:** Interpretation of Kendall rank correlation coefficient values.

**The value of W**	**Interpretation**	**Confidence in the interpretation of factors**
0.1	Very weak consensus	Does not exist
0.3	Poor consensus	Low
0.5	Medium consensus	Moderate
0.7	Strong consensus	High
0.9	Very strong consensus	Very high

The value of such scale equals to one and zero when there is and is not complete coordination or agreement, respectively (44). Two statistical criteria are presented to decide whether to stop or continue Delphi rounds. The strong consensus among panel members is considered as the first criterion, which is determined by the Kendall rank correlation coefficient. The persistence of the coefficient or its slight growth in two consecutive rounds in the absence of consensus indicates that there has been no increase in the agreement among the members and the polling process should be stopped. It is worth noting that the statistical significance of the Kendall coefficient does not suffice to stop the Delphi process. Even small Kendall values are regarded as significant for panels with more than 10 members (43).

Therefore, the study entered the third round in order to raise the consensus of experts on the subject. The members of the panel were once again asked to comment on the identified effects and identify their significance during this stage. The experts who refused to send the questionnaire were excluded from the process and analyzes were considered based on the final answers. We asked 30 experts to answer the questions. But 8 of them were removed due to non-participation and finally 22 people participated in completing the questionnaires. Table 1 shows the participation of each expert in each round.

## Results

### Effects of the COVID-19 pandemic on ecotourism status from experts' point of view

As indicated, 37 effects of the COVID-19 on ecotourism status were identified during the pre-Delphi stage, of which 13 were removed in the validation stage and 24 remained. Then, the experts were asked to categorize the factors identified in the literature review including the decreased number of incoming tourists, the reduced activity of hotels and resorts, the declined income of goods and service suppliers for tourists, the decreased activity of travel agencies and the tourist tours, as well as positive and negative environmental effects.

During the first round of the Delphi stage, the panel of experts was asked to identify the effects of the COVID-19 on the ecotourism status in the study area not indicated in the literature review in addition to highlighting the significance of the remaining effects (24 cases). The experts added five more cases to indicated ones and a total of 29 effects were identified to form the basis of the second round (Table 3).

**Table 3:** Identified effects of the COVID-19 pandemic on ecotourism status.

**Factors**	**Category**	**Delphi rounds**					
		**Round 1**		**Round 2**		**Round 3**	
		**Factor inclusion**	**Mean**	**Mean**	**W**	**Mean**	**W**
Number of tourists (4.90)	Decreased number of tourists entering the ecotourism areas of the province	√	4.35	4.20	0.83	4.95	0.89
	Decreased number of tourists entering the Lorestan province	√	4.60	4.70	0.87	4.85	0.90
Activity of hotels and accommodations (4.81)	Decreased number of travelers applying to use eco-lodges	×	-	4.60	0.73	4.90	0.77
	Decreased number of travelers applying to use the guest house	×	-	4.70	0.85	4.85	0.88
	Decreased number of travelers applying to use the hotel	√	4.55	4.50	0.88	4.80	0.91
	Adjusting the manpower by hotels	√	4.55	4.60	0.72	4.70	0.79
Income of suppliers of tourist goods and services (4.04)	Decreased income of restaurants in ecotourism areas	√	4.55	4.70	0.81	4.60	0.89
	Decreased income of contractors of service companies located in ecotourism areas including cleaning companies, parking lots, and the like	√	4.30	4.45	0.90	4.55	0.92
	Decreased income of tourist entertainment jobs including children's play equipment and the like	√	4.40	4.60	0.86	4.55	0.90
	Decreased sales of local conversion and complementary industries in ecotourism areas	√	4.20	4.30	0.69	4.40	0.75
	Decreased sales of handicrafts in ecotourism areas	√	4.30	4.45	0.84	4.40	0.89
	Decreased income of food sellers including supermarkets and chain stores in ecotourism areas	×	-	4.15	0.71	4.10	0.77
	Decreased income of locals living in ecotourism areas	√	3.90	3.85	0.69	3.80	0.76
	Adjusting the manpower by service companies located in ecotourism areas	√	3.50	3.45	0.83	3.35	0.86
	Decreased income of jobs such as peddling in ecotourism areas	×	-	3.35	0.88	3.20	0.91
Activities of travel agencies and tourist tours (3.81)	Decreased income of tour companies	√	4.45	4.60	0.80	4.65	0.87
	Unemployment of tour guides	√	4.35	4.40	0.89	4.30	0.94
	Decreased ticket sales by ground public transport terminals	√	3.45	3.40	0.79	3.45	0.82
	Adjusting the manpower by tour companies	√	3.45	3.30	0.75	3.35	0.80
	Decreased ticket sales by airline travel agencies	√	3.20	3.15	0.88	3.25	0.90
Positive environmental effects (3.59)	Reduced pollution including waste and the like in forest and natural areas due to decreased number of tourists entering the forest areas	√	4.00	4.10	0.90	4.30	0.91
	Reduced deforestation due to decreased number of tourists entering the forest areas	√	4.00	4.05	0.87	4.20	0.91
	Reduced disease due to restrictions on human contact during tourism	√	3.50	3.45	0.83	3.55	0.88
	Reduced air pollution due to decreased travel	√	2.90	3.10	0.78	3.20	0.82
	Creation of an appropriate opportunity for successful reproduction of endangered species in the absence of tourists	×	-	2.50	0.75	2.70	0.76
Negative environmental effects (3.43)	Increased poaching and deforestation due to reduction of tourism revenues in indigenous communities	√	3.85	3.75	0.90	3.70	0.93
	Reduced oversight by government and forest protection agencies during pandemics and more possibility for timber smuggling by local people	√	3.35	3.45	0.73	3.30	0.77
	Reduced oversight by government and forest protection agencies during pandemics and more possibility for poaching	√	3.30	3.40	0.87	3.30	0.90

√ Factors remaining in the pre-Delphi stage and × Factors identified in the first round of Delphi.

During the second round of the Delphi stage, the experts were asked to determine the significance of the 29 identified effects. In addition, the Kendall rank correlation coefficient was calculated for all of the factors, indicating that none of the factors were eliminated. However, the study entered the third round of the Delphi stage for stronger consensus (Table 3), in which the significance of the factors was determined once again and Kendall rank correlation coefficient significantly improved with a strong consensus.

The effects of the COVID-19 pandemic on ecotourism status in the study area were categorized as follows (Table 3).

### The number of incoming tourists

The overall average of this category equals to 4.90 with higher score than the other five categories. In other words, the COVID-19 pandemic affects the number of incoming tourists significantly. The most significant consensus among the experts on the effects of the COVID-19 pandemics was as follows; the decreased number of tourists entering the ecotourism areas of the province with an average of 4.95 and the decreased number of tourists entering the Lorestan province with an average of 4.85.

### Activities of hotels and resorts

Effect on the activity of hotels and resorts was identified as the second category of the COVID-19 effects on ecotourism with an overall average of 4.81. The effects identified in this category are prioritized as follows; the decreased number of travelers applying to use eco-lodges with an average of 4.90, the decreased number of travelers applying to use the guest house with an average of 4.85, the decreased number of travelers applying to use the hotel with an average of 4.80 and the adjusting manpower by hotels with an average of 4.70.

### Income of goods and service suppliers for tourists

Here, the highest consensus among the experts on the effects of the COVID-19 on ecotourism is related to the income of goods and service suppliers for tourists with an overall average of 4.04. According to the average, the order of significance for the effects in this category is as follows; the decreased income of restaurants in ecotourism areas with an average of 4.60, the decreased income of contractors of service companies located in ecotourism areas including cleaning companies, parking lots, and the like with an average of 4.55, the decreased income of tourist entertainment jobs including children's play equipment and the like with an average of 4.55, the decreased sales of local conversion and complementary industries in ecotourism areas with an average of 4.40, the decreased sales of handicrafts in ecotourism areas with an average of 4.40, the decreased income of food sellers including supermarkets and chain stores in ecotourism areas with an average of 4.10, the decreased income of locals living in ecotourism areas with an average of 3.80, adjusting the manpower by service companies located in ecotourism areas with an average of 3.35 and the decreased income of jobs such as peddling in ecotourism areas with an average of 3.20.

### Activities of travel agencies and tourist tours

Here, the highest consensus among the experts on the effects of the COVID-19 on ecotourism is related to the activities of travel agencies and tourist tours with an average of 3.81. According to the average obtained for each effect, the prioritization of this category is as follows; the decreased income of tour companies with an average of 4.65, the unemployment of tour guides with an average of 4.30, the decreased ticket sales by ground public transport terminals with an average of 3.45, adjusting the manpower by tour companies with an average of 3.35 and the decreased ticket sales by airline travel agencies with an average of 3.25.

### Positive environmental effects

Positive environmental effects are defined as the fifth category with an average of 3.59. The effects identified in this category are prioritized as follows: reduced pollution including waste and the like in forest and natural areas due to decreased number of tourists entering the forest areas with an average of 4.30, reduced deforestation due to the decreased number of tourists entering the forest areas with an average of 4.20, reduced disease due to restrictions on human contact during tourism with an average of 3.55, reduced air pollution due to decreased travel with an average of 3.20 and the creation of an appropriate opportunity for successful reproduction of endangered species in the absence of tourists with an average of 2.70.

### Negative environmental effects

Such effects are defined as the least significant category with the lowest total average of 3.43. According to the average, the order of significance for the effects in this category is as follows; increased poaching and deforestation due to the reduction of tourism revenues in indigenous communities with an average of 3.70, reduced oversight by government and forest protection agencies during pandemics and more possibility for timber smuggling by local people with an average of 3.30 and the reduced oversight by government and forest protection agencies during pandemics and more possibility for poaching with an average of 3.30.

## Discussion

The effects of the COVID-19 pandemic on ecotourism status in the study area were categorized as follows (Table 3).

### The number of incoming tourists

Experts have probably given this category a higher score since the decrease in the number of incoming tourists is considered as the most direct effect of the COVID-19 pandemic. In other words, the decrease in the number of incoming tourists is regarded as the first and most noticeable effect when something unusual occurs such as the pandemic of a disease. The results confirm findings of Mudzengi et al. (35) and Stone et al. (30). Based on the prioritization of the two effects in this category, the decrease in the number of tourists entering the ecotourism areas has been scored more than the decrease in the number of incoming tourists, meaning that ecotourism is considered as more significant in Lorestan province than other types of tourism, resulting in feeling the decrease in the number of tourists entering the ecotourism areas more than other types of tourism during the COVID-19 pandemic. It is suggested that in order to prevent the decrease of tourists, the authorities should plan so that tourists gradually enter the ecotourism areas since a large number of people should not gather at a specific time and place due to the significance of their health during the pandemic. The Tourism Office should schedule a continuous and gradual arrival in coordination with tourist tours. Gabriel-Campos et al. (36) also emphasize on the management and gradual entry of tourists to ecotourism areas during epidemics such as Covid-19.

### Activities of hotels and resorts

Based on this classification, the COVID-19 has had the greatest effect on the activity of hotels and resorts after decreasing the number of tourist arrivals, which is regarded as its most noticeable effect on tourism. Some studies Stone et al. (30) have reported declining the incomes of hotels and resorts during the COVID-19 pandemic. Prioritizing the items in the aforementioned category is considered as a noteworthy point. According to the prioritization, the COVID-19 pandemic has reduced the number of travelers in eco-lodges, guest houses, and hotels, respectively, indicating that eco-lodges are the most used among tourists in ecotourism areas and crises such as the COVID-19 reduce the number of travelers to this type of accommodation more than others. According to the result that tourists in Lorestan province use more eco-lodges, it is suggested to provide more support for the establishment of these residences.

### Income of goods and service suppliers for tourists

The third category of effects identified in order of importance is the impact of Covid-19 on the income of goods and service suppliers for tourists. The study results are in line with Stone et al. (30). Restaurants, service companies such as cleaning companies, parking lots, and the like, tourist entertainment jobs, local conversion and complementary industries, handicrafts, and food stores, as well as local people offer goods and services to tourists in ecotourism areas, indicating that those who cannot offer their goods and services in the absence of tourists and have no other alternative have suffered the most financial loss due to the COVID-19 pandemic and absence of tourists. Local people or vendors are less affected by the COVID-19 and the decline in the number of tourists since such people usually find alternative markets, while jobs such as restaurants or service companies located in ecotourism areas have suffered the most. Hosseini et al. (14) believe that it is very important to find solutions for affected businesses during the coronavirus pandemic. So, it is suggested that the owners of these businesses turn to online sales to prevent the income of tourism sector activists from decreasing. It is necessary for these people to see internet marketing training.

### Activities of travel agencies and tourist tours

The reduction in the activities of travel agencies and tourist tours has been identified as the fourth category of effects. These results have been confirmed by Foo et al. (32), which is not regarded as strange at all although the ranking results of the items in the above-mentioned category show that the income and adjustment of the tourist tour staff and the travel agencies are affected by the COVID-19 pandemic, respectively because not all travelers to travel agencies are not considered as tourists. Thus, the aforementioned agencies are affected by the COVID-19 less than tourist tours, and all of their activities depend on the presence of tourists. It is suggested that in order to avoid reducing the income of tourist agencies and tour guides, the activists of this sector should offer virtual visits to ecotourism destinations during the outbreak of pandemics. This type of tourism has gained many fans now. As mentioned, Hosseini et al. (14) have emphasized the adaptation of affected businesses during the coronavirus pandemic.

### Positive environmental effects

The next category of the effects of Covid-19 is the positive effects of this pandemic on ecotourism. The study results are in line with Buckley (31) and Jovanović et al. (12). These researchers indicated some positive effects of the COVID-19 on the environment, despite its negative effects on the ecotourism sector. The natural positive effects in the absence of tourists may be attributed to inappropriate and destructive environmental behaviors of some tourists in nature. According to some studies, nature-destroying behaviors occur less in the absence of some tourists. Pollution in the air and natural environment has decreased, fewer trees have been cut down, and even endangered species have had the opportunity to reproduce. In fact, such result should make tourists think that more kindness to ecotourism destinations is needed after the end of the COVID-19 and return to nature. It is suggested to give environmental education to tourists, so that tourists have better environmental behaviors with ecotourism destinations after the end of Covid-19.

### Negative environmental effects

The negative effects of the COVID-19 on the environment should be considered, despite the positive ones. Care in the cases in this category shows that the occurrence of the negative effects results from reduced oversight of government and conservation organizations for nature protection since the COVID-19 has decreased the possibility of visiting and monitoring ecotourism areas by the above-mentioned organizations, indicating that environmental education to people to strengthen their environmental attitudes and behaviors toward nature has not been sufficient or has failed to have the required quality because the timber smuggling by local people and poaching have increased in the absence of conservationists and government officials. The study results are in line with Amador-Jiménez et al. (28), Cherkaoui et al. (27), Lendelvo et al. (29), Mudzengi et al. (35) that indicated the negative environmental effects of the COVID-19, as well. Environmental education to local people and more monitoring to protect natural areas during pandemics are other suggestions of this research.

## Conclusion

The COVID-19 has affected the tourism sector, particularly ecotourism significantly. The COVID-19 has affected the ecotourism both positively and negatively although its effects on other parts are mainly negative. The present study sought to investigate both types of effects completely. Lorestan province in Iran is regarded as an ecotourism destination for a large number of tourists due to its various ecotourism areas. Therefore, this study aimed to review the effects of the COVID-19 pandemic on its ecotourism status. Thus, 29 effects of the COVID-19 pandemic on ecotourism status were identified utilizing Delphi technique.

Based on the results, the effects were divided into six categories including decreased number of incoming tourists, reduced activity of hotels and resorts, declined income of goods and service suppliers for tourists, decreased activity of travel agencies and tourist tours, as well as positive and negative environmental effects.

New strains of the COVID-19 may be released worldwide in the coming years, meaning that the end of the COVID-19 epidemic crisis cannot be predicted. Thus, in order to reduce the negative effects of the COVID-19, the tourism sector should apply the necessary strategies to return to a more normal state like other sectors of the economy. In other words, the managers in ecotourism destinations should take measures to ensure greater compatibility between tourism activities and the spread of the virus. Such measures can be identified through further studies and according to the effects identified. Therefore, assessing the mechanisms for the acceptance of the COVID-19 epidemic by tourists during tourism activities can be the future research directions. Identifying mechanisms that increase the prior preparation of ecotourism destination managers during epidemic outbreaks is another suggestion of this study for researchers in the future. Evaluating the solutions which help the tourism managers provide better tourism services during the COVID-19 pandemic can be useful, as well. Also, knowing the types and content of environmental education for tourists during epidemics is another suggested topic for the future.

The final point refers to the limitations of the research. In this research, like many researches that have been conducted during the COVID-19 epidemic, we were also faced with the poor cooperation of the statistical community to collect data. To overcome this problem, we had to complete the questionnaires online.

## Data availability statement

The datasets presented in this study can be found in online repositories. The names of the repository/repositories and accession number(s) can be found in the article/supplementary material.

## Author contributions

MR: methodology, software, and writing—original draft preparation. MM: conceptualization and data curation. HZ: supervision and validation. All authors contributed to the article and approved the submitted version.

## Funding

This article was derived from a research project and was done with the financial support of Lorestan University.

## Conflict of interest

The authors declare that the research was conducted in the absence of any commercial or financial relationships that could be construed as a potential conflict of interest.

## Publisher's note

All claims expressed in this article are solely those of the authors and do not necessarily represent those of their affiliated organizations, or those of the publisher, the editors and the reviewers. Any product that may be evaluated in this article, or claim that may be made by its manufacturer, is not guaranteed or endorsed by the publisher.
